# Infographic: Positioning In Macular hole Surgery (PIMS) trial

**DOI:** 10.1038/s41433-021-01559-1

**Published:** 2021-05-26

**Authors:** Nikolaos Tzoumas, Declan C. Murphy, Alexander Mehta, Islam Mostafa, Salman N. Sadiq, Anna Song, Mohaimen Al-Zubaidy, Ali E. Ghareeb, David H. Steel

**Affiliations:** Biosciences Institute, Newcastle University, Newcastle upon Tyne, UK

**Keywords:** Outcomes research, Surgery

**Fig. 1:**
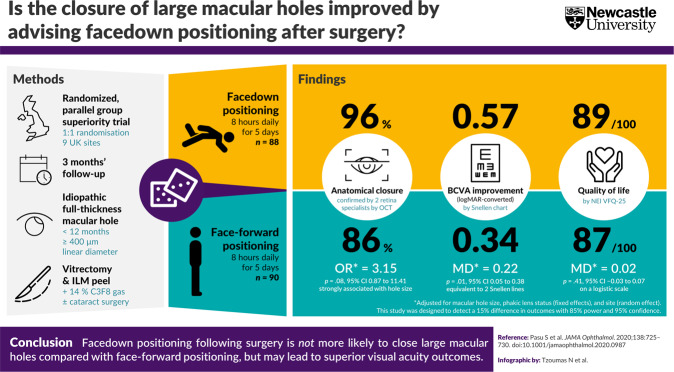
The Positioning In Macular hole Surgery (PIMS) trial showed that facedown positioning following surgery is not more likely to close large full-thickness macular holes compared to face-forward positioning, but may result in a modest benefit in visual acuity outcomes at three months. BCVA best-corrected visual acuity, C3F8 perfluoropropane, CI confidence interval, ILM inner limiting membrane, MD mean difference, NEI VFQ-25 National Eye Institute Visual Function Questionnaire 25, OCT optical coherence tomography, OR odds ratio, UK United Kingdom.

